# The lower limit margin: a determinant of autoregulatory sensitivity to changes in cerebral perfusion pressure

**DOI:** 10.1186/s13054-025-05686-z

**Published:** 2025-10-27

**Authors:** Stefan Yu Bögli, Ihsane Olakorede, Erta Beqiri, Xuhang Chen, Andrea Lavinio, Peter Hutchinson, Peter Smielewski

**Affiliations:** 1 Brain Physics Laboratory, Division of Neurosurgery, Department of Clinical Neurosciences, University of Cambridge, Cambridge, UK; 2 Division of Neurosurgery, Department of Clinical Neurosciences, University of Cambridge, Cambridge, UK; 3 Division of Anaesthesia, Department of Medicine, University of Cambridge, Cambridge, UK; 4 Department of Neurology and Neurocritical Care Unit, Clinical Neuroscience Center, University Hospital Zurich, University of Zurich, Zurich, Switzerland

**Keywords:** Traumatic brain injury, Multimodality monitoring, Cerebral perfusion pressure, Cerebrovascular autoregulation

## Abstract

**Introduction:**

The Lower Limit Margin, defined as the difference between the autoregulation-informed optimal cerebral perfusion (CPP) target (CPPopt) and the lower limit of autoregulation (LLA), was recently introduced as a potential dynamic prognostic marker after traumatic brain injury (TBI). Conceptually, CPPopt marks the CPP associated with “optimal” autoregulatory function, while LLA represents the CPP at which cerebrovascular autoregulation is already impaired and deteriorates with further decreases in CPP. Based on the hypothesis that the effect of the Lower Limit Margin on outcome is critically mediated by the level of short-term CPP variability and the associated increase in time spent below the LLA, we aimed to explore the prognostic value of this novel marker.

**Materials and methods:**

In a prospective cohort of 234 severe TBI patients receiving invasive multimodality monitoring, we evaluated the prognostic and physiological relevance of the Lower Limit Margin. Minute-by-minute CPP, CPPopt, and LLA were derived using automated, validated methods. The association between the Lower Limit Margin with 6-month Glasgow Outcome Scale, short-term CPP variability, and autoregulatory burden (time spent below LLA) was assessed using logistic regression, ordinal analyses, mixed-effects models, causal mediation analysis, and generalized additive modeling.

**Results:**

Patients with wider Lower Limit Margins experienced significantly less time with CPP below the LLA (β = − 2.1, CI − 2.2 to − 1.9) and had decreased odds of unfavorable outcome (OR 0.59, CI 0.41–0.83, *p* = 0.003). Causal mediation analysis indicated that 58.9% effect of the Lower Limit Margin on outcome was mediated by time spent below the LLA. A significant interaction between Lower Limit Margin and short-term CPP variability was observed: a narrow Lower Limit Margin combined with high short-term CPP variability were associated with the worst outcomes and higher amount of time spent with CPP below LLA. Nearly 50% of hourly short-term CPP variability exceeded ± 5 mmHg.

**Conclusion:**

Our findings demonstrate that a narrow Lower Limit Margin is a marker of increased physiological vulnerability, associated with more frequent and severe CPP insults and worse neurological outcomes after TBI. These results support a shift away from single-threshold strategies (e.g., CPPopt or LLA alone) toward a resilience-informed approach that integrates the dynamic interplay between autoregulatory reserve and short-term CPP variability.

**Supplementary Information:**

The online version contains supplementary material available at 10.1186/s13054-025-05686-z.

## Introduction

Cerebral perfusion pressure (CPP) management is one of the key aspects of intensive care management after traumatic brain Injury (TBI) [1]. While current guidelines still propose fixed targets ranging between 60 and 70 mmHg [1], personalized CPP targets, which account for the state of cerebrovascular autoregulation, have largely outperformed these when assessed against outcome [2–5]. Cerebrovascular autoregulation describes an innate mechanism that aims at stabilizing cerebral blood flow [6]. Specifically, it counteracts slow changes in systemic driving pressure by adjusting arteriolar diameter [6]. In intensive care, different personalized targets have been proposed for the management of TBI patients, namely CPPopt (i.e., optimal CPP) [3] and the LLA (i.e., the lower limit of autoregulation) [4, 5]. Both are based on the pressure reactivity index (PRx), a proxy measure of cerebrovascular autoregulation which quantifies the change in intracranial pressure (ICP – herein used to quantify the magnitude of change in diameter of the arterioles) to slow changes in arterial blood pressure (ABP) [7]. First described in 2002 [3], CPP and PRx often display a distinct U-shaped relationship, from which both CPPopt and LLA are derived. Specifically, CPPopt is defined as the CPP value associated with the lowest PRx across this relationship, while LLA is the CPP value at which cerebrovascular autoregulation is already impaired and further deteriorates with decreasing CPP [2–5]. Particularly, below LLA, cerebral blood flow cannot be stabilized sufficiently, leading to the risk of hypoxia and hypoperfusion [8, 9]. Of note, PRx and PRx derived metrics reflect cerebrovascular reactivity [7], which in itself is just one aspect affected by the cerebrovascular autoregulation mechanism [5]. However, given that PRx remains the most widely used continuous proxy for the patients autoregulatory status, the term LLA and autoregulation (rather than lower limit of reactivity and cerebrovascular reactivity) will be used throughout for simplicity.

Both CPPopt and LLA can be estimated at the bedside using automated methods [2, 10, 11]. From a conceptual standpoint [12], when CPP is maintained at CPPopt, the cerebrovascular autoregulation mechanism “optimally” supports the stabilization of cerebral blood flow. Conversely, LLA is more frequently seen as a critical “safety” threshold, since a higher frequency of CPP below the LLA is strongly associated with worse outcomes due to the association to decreased cerebral blood flow [5]. Most recently, we introduced the Lower Limit Margin, which describes the difference in CPP between CPPopt and LLA [13]. The Lower Limit Margin was distinctly dynamic throughout the TBI course, with patients with narrow Lower Limit Margins displaying distinctly worse outcomes. Of note, in some patients, the Lower Limit Margin was as narrow as 5–6 mmHg. This raises the question of whether the pragmatically used margin of ± 5 mmHg around CPPopt should be revisited.

In the present analysis, we hypothesize that the effect of the Lower Limit Margin on outcome is mediated by the time spent below the LLA, which is likely dependent on the innate variability in CPP that each patient displays. Specifically, a narrow Lower Limit Margin may amplify the effects of short-term CPP variability on outcome by increasing the risk of CPP dropping below the LLA, thereby contributing to secondary ischemic injury and worse functional outcomes in patients with severe TBI. Through this analysis, we seek to advance the understanding of autoregulation-guided management and work towards extending the concept of individualised therapeutic CPP targets to individualised therapeutic ranges.

## Materials and methods

We accessed de-identified TBI records from the Brain Physics Lab research database, which was approved by the Research Ethics Committee (REC 23/YH/0085). The dataset includes high-resolution monitoring data and clinical descriptors from consecutive TBI patients admitted to the Neurocritical Care Unit at Addenbrooke’s Hospital, Cambridge University Hospital NHS Foundation Trust, University of Cambridge [14]. The patients continuously receive monitoring as part of routine care for the duration of their stay, if deemed necessary (i.e., monitoring is stopped if the patient receives redirection of care to palliative measures or if the patient has been sufficiently stabilized, is clinically assessable, and no further secondary injuries are foreseeable). No study-specific data were collected, and therefore, the requirement for informed consent was waived. The management of the patients largely follows the Brain Trauma Foundation guidelines [1]. The adapted local guidelines have previously been described [15]. Of note, while targeting CPPopt is part of the advised Tier 1 ICP/CPP management, it is still up to the treating physician whether such a target should be followed.

### Study population

Consecutive patients admitted between 06/2021 and 12/2023 were evaluated for inclusion. Inclusion criteria were: (1) Acute TBI with invasive ICP monitoring (using an intraparenchymal ICP pressure transducer); (2) Available 6-month outcome (Glasgow Outcome Scale – GOS [16]). There were no specific exclusion criteria. The following clinical data was extracted from the database: sex, age, Glasgow Coma Scale (GCS) [17], pupillary reactivity (both reactive vs. one reactive vs. none reactive), presence of intracranial bleed (extradural, intracerebral, or subdural hematoma, contusion, traumatic subarachnoid hemorrhage), presence of extracerebral injuries (divided into injuries to thorax, abdomen, extremities, pelvic, skull, or spine), presence of isolated TBI (no extracranial injuries), decompressive craniectomy (DC), and GOS. GOS was assessed 6 months after ictus during outpatient consultations or via telephone interviews by trained staff. Outcome was evaluated assessing the ordinal scale (good recovery vs. moderate disability vs. severe disability vs. dead/vegetative representing GOS 5 vs. 4 vs. 3 vs. 2/1) or as dichotomized outcome (GOS 1–3 vs. 4–5).

### Monitoring data acquisition and preprocessing

High resolution (i.e., waveform, 240 Hz) physiological data were collected in real time at the bedside from GE Solar and subsequently Carescape B650 monitors (General Electrics, Massachusetts, USA), and pre-processed using the ICM+ software [18] (Cambridge Enterprises, University of Cambridge, UK). ICP was measured using intraparenchymal pressure transducers (Codman ICP MicroSensor, Codman & Shurtleff, Raynham, Massachusetts). ABP was measured using arterial lines (Baxter Healthcare, Deerfield, Illinois, USA) inserted into the radial or femoral artery and zeroed at the level of the foramen of Monro (as estimated by tragus level). Raw ABP and ICP signals were curated as described in detail elsewhere [14] to remove the following artifacts: sections with arterial line failure, readings outside of physiological ranges or without a pulse, and readings of artifactual nature (high spectral edge frequency). Missing data resulting from artifact clearing was not imputed. Given the lack of validated imputation methods for high-resolution multimodality neuromonitoring, we retained only the available cleaned data. Both PRx and CPPopt calculations are robust to gaps, as relatively large sections of missing data can be tolerated before calculations become invalid (see subsequent sections).

**Fig. 1:**
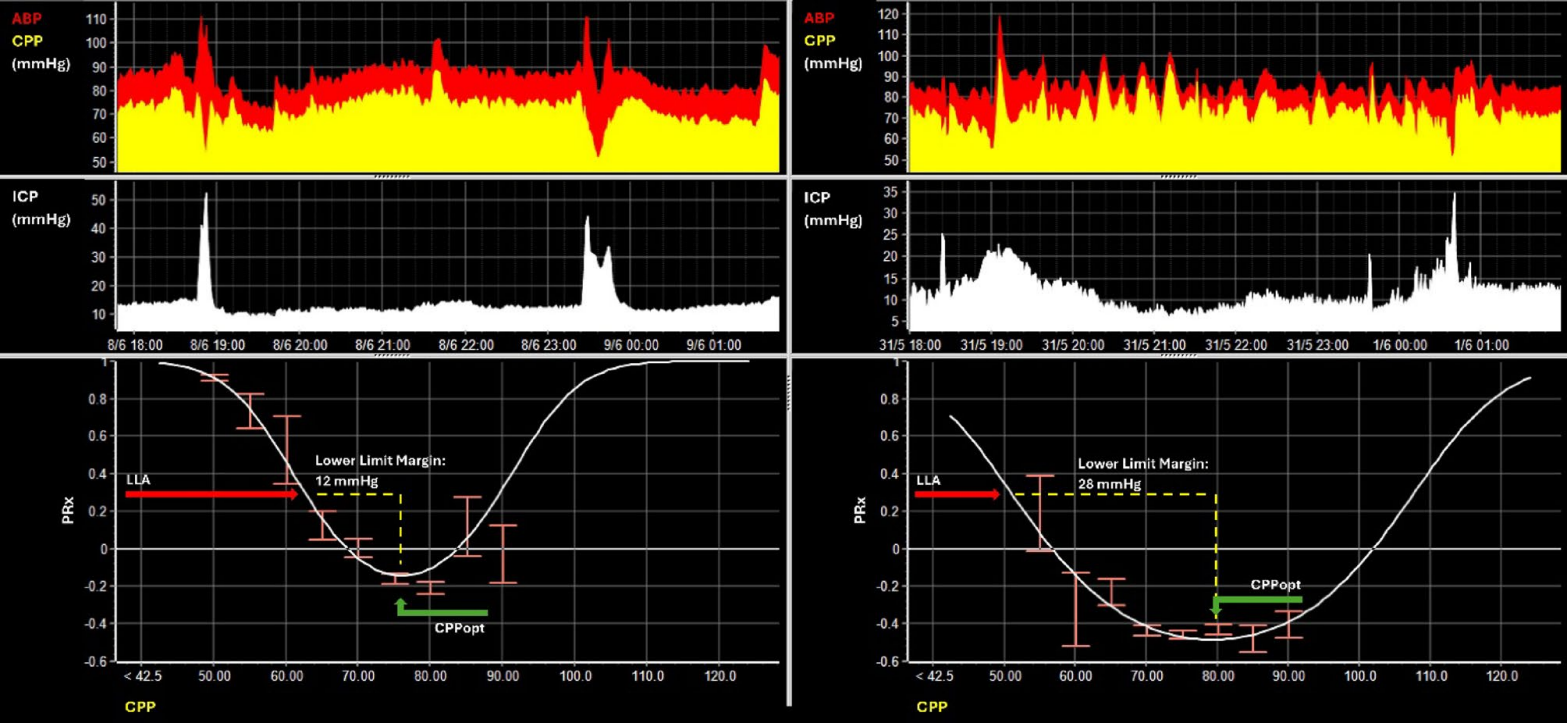
Estimating CPPopt, LLA, and the Lower Limit Margin**.** Two example 8-hour time trends of ABP and CPP (top), ICP (middle), and the corresponding CPP/PRx relationships (bottom) from the same patient are shown. Building on the original description in 2002[3], as well as later iterations [10, 19], CPPopt can be estimated dynamically. In simplified terms, PRx is first calculated as described in the main manuscript and plotted against CPP (bottom charts). Then, to derive CPPopt and LLA, a second order polynomial is fitted to the means of binned, fisher transformed, PRx and CPP data considering either the full available data set (as illustrated here) or using more complex (and consequently not easily visualized) methods such as the multi-window weighted approach. For the latter, the PRx-CPP relationship is evaluated for multiple overlapping time windows of CPP/PRx data, where only parabolic fits are accepted and their contribution is weighted according to curve fit quality, the shape of the fitted curve portion and the window duration, with results combined through an exponentially weighted average [10, 11]). Within the figure, the resulting fit is shown in a form of the white parabolic curve. Within the resulting curves, CPPopt is defined as the CPP corresponding to the nadir of this curve, while LLA (technically a lower limit of reactivity LLR) represents the lower breakpoint where cerebrovascular autoregulation becomes decisively impaired (reflected by a PRx of 0.3) and progressively deteriorates further with additional decreases in CPP. The corresponding locations of LLA and CPPopt from which the Lower Limit Margin is derived are marked using red and green arrows. The Lower Limit Margin simply describes the distance between these two autoregulation indicators. When comparing the two curves at the bottom, distinctly different Lower Limit Margins can be appreciated with a margin of 12 mmHg for the section on the left and a margin of 28 mmHg for the section on the right

PRx was calculated by computing the moving correlation coefficient between consecutive 10-second mean ABP and ICP values over 5-minute intervals, with a missing data limit of 50% per data window [7]. CPPopt and LLA were estimated automatically every minute from the past 8-hour data windows as described previously, by fitting a parabolic curve to 5-minute median CPP and PRx values using the multi-window weighted approach [10]. Similar to the missing data limit of PRx, the corresponding missing data limit for the estimation of CPPopt is 75% (i.e. there needs to be at least 2 h of consecutive multimodality monitoring data for the calculation of CPPopt and LLA). The PRx cutoff for LLA was chosen to be 0.3. The Lower Limit Margin was calculated as the difference between CPPopt and LLA on a minute-by-minute basis. The methodology used the calculation of CPPopt and LLA, as well as examples of a narrow and a wide Lower Limit Margin are shown in Fig. 1. For the subsequent analyses, minute-by-minute averages were used. Three metrics were used to describe autoregulatory insults, specifically describing Dose (area under the curve above (for CPPopt) or below (for CPPopt or LLA)), hourly dose (hDose; total dose divided by hours of valid data), and percentage time (ptime; percentage of valid data, during which CPP was above or below CPPopt or LLA). Short-term CPP variability was quantified by extracting the standard deviation of each consecutive non-overlapping hour of data. For the statistical analyses described, we used overall averages unless otherwise specified.

### Statistical analysis and visual exploration

Statistical analyses and figure preparation were performed using R (R: A language and environment for statistical computing, version 4.4.1; the specific packages used were *tidyverse*,* dplyr*,* ggplot2*,* MASS*,* mgcv*,* mediation*,* lme4*). The statistical significance was set at the level of 0.05. No adjustment for multiple testing was applied due to the exploratory nature of the study. The data was analyzed focusing on two distinct lines of investigation, first assessing the Lower Limit Margin as a distinct prognostic marker and second, assessing the intricate relationship between Lower Limit Margin, short-term CPP variability, increases in time spent below the LLA, and functional outcome.

For the first section, the methods described largely follow the initial description from the previous paper that introduced the Lower Limit Margin [13]. Univariable analysis was performed using logistic regression comparing patients with favorable vs. unfavorable outcome, respectively. Additionally, to examine how the Lower Limit Margin evolves over time when considering the GOS, we constructed mixed effects models using daily Lower Limit Margin values and day post injury as fixed effects and patient ID as a random effect. Of note, since CPPopt and derived metrics such as the Lower Limit Margin are derived by assessing the prior 8 h of data, each daily average incorporates a small portion of data from the prior day. This method is consistent with previous research and is based on the hypothesis that the current state of autoregulation can be derived from the behavior displayed within the last hours. Given the ordinal nature of the GOS outcome, we also performed two ordinal regression analyses [20]. Proportional odds logistic regression was used to determine a common odds ratio reflecting the average shift in odds across the full ordinal scale when assessed across shifting thresholds (i.e., between Dead/Vegetative and Severe Disability, between Severe Disability and Moderate Disability, between Moderate Disability vs. Good Recovery). The proportional odds assumption was not formally tested, as the primary aim was to assess the overall direction and magnitude of association across the ordinal outcome scale rather than providing evidence that this change is the same for each cutoff [21]. Sliding dichotomy was used to assess whether patients suffer a worse outcome than expected (based on the initial clinical presentation) [20]. For each patient, based on the baseline covariates – age, sex, motor GCS, pupillary reactivity, type of hemorrhage, isolated TBI vs. polytrauma, extracranial injury (to the abdomen, extremities, pelvis, skull, spine, or thorax separately), and DC – a prognostic risk probability for unfavorable outcome was estimated. The resulting scores were then divided into three prognostic groups of roughly equal size corresponding to low, intermediate, and high likelihood of unfavorable outcome. For each prognostic group a separate cutoff was defined to dichotomize outcome into favorable and unfavorable, with the adjusted favorable outcome classified as: GOS 5 for the group with low likelihood for unfavorable outcome, GOS 4–5 for the group with intermediate likelihood for unfavorable outcome, and GOS 3–5 for the group with high likelihood for unfavorable outcome. The resulting baseline severity-adjusted outcome variable was then assessed against the Lower Limit Margin using logistic regression. For both methods, bootstrapping was applied for internal validation and to acquire the 95% confidence interval (CI). To explore the Lower Limit Margin further, we calculated the correlation coefficients between absolute averages of the Lower Limit Margin and different clinical and monitoring metrics. Pearson correlation was used for continuous variables and Spearman correlation for ordinal metrics. Lastly, to investigate the Lower Limit Margin further, we performed a linear regression analysis between the regularly assessed clinical parameters and the Lower Limit Margin width to assess to what extent the regularly assessed clinical parameters can be used to predict the Lower Limit Margin width.

For the second analysis, we were interested in assessing the relationship between the Lower Limit Margin, short-term CPP variability (quantified using standard deviation for each consecutive non-overlapping hour of data), and the percentage time spent below the LLA. For the following analyses, hourly or daily averages were extracted. In a first step, overall frequencies of hourly short-term CPP variability across the data were explored visually. Then, to model the non-linear joint effects of Lower Limit Margin and short-term CPP variability on the probability of unfavorable outcome, a generalized additive model was fitted to daily averages using thin plate spline regression with the patient ID and day added as nested random effects. Specifically, a binary logistic model with a logit link function was used to estimate the probability of unfavorable outcome based on a two-dimensional smooth term incorporating both Lower Limit Margin and short-term CPP variability. A random intercept for patient ID was included to account for repeated measurements. For visualization, a risk surface plot was generated, illustrating the predicted probabilities of unfavorable outcome across the observed combinations of Lower Limit Margin and short-term CPP variability. In a second step, to explore the hypothesis that the effect of Lower Limit Margin and short-term CPP variability on outcome is mediated by the time spent below LLA, the following additional analyses were performed. First, a causal mediation analysis [22] was performed to evaluate whether the effect of Lower Limit Margin width on outcome was mediated through the percentage time spent below the LLA. For this purpose, we first applied a linear regression model to estimate the effect of the Lower Limit Margin on the mediator. Second, a logistic regression model was used to model the effect of both the Lower Limit Margin and the mediator on outcome. Lastly, a mediation analysis was applied to estimate the average causal mediation effect, the average direct effect, the total effect, and the proportion of the total effect mediated. Confidence intervals were derived using nonparametric bootstrapping with 1000 simulations. Second, a linear mixed-effects model was employed based on daily averages with the percentage time below LLA as the dependent variable, the Lower Limit Margin and short-term CPP variability as the fixed effects, and the patient ID and day post-injury as random effects to account for repeated measures and potential clustering within patients.

## Results

A total of 234 consecutive patients with TBI were included in the analysis. The inclusion/exclusion flow chart has previously been presented [23]. The patient characteristics are described in Table 1. The median age was 45 years (IQR 35–65), and most patients were male (75%). The median initial Glasgow Coma Scale was 4 (IQR 1–5), and the majority of patients had sustained an isolated TBI (23%). At 6 months, outcome distribution according to the GOS was as follows: good recovery (GOS 5) in 13%, moderate disability (GOS 4) in 29%, severe disability (GOS 3) in 28%, and death or persistent vegetative state (GOS 1,2) in 30%. Overall, 45,200 h of monitoring data were assessed, corresponding to a median of 157 (IQR 88–279) hours per patient. The distribution and temporal coverage of CPP stratified by outcome category are detailed in Supplement A. No differences in monitoring length depending on the outcome category could be identified. The median yield of CPPopt and LLA during the overall monitoring period was 81% (IQR 74–86).

**Table 1:** Patient characteristics*

Characteristic	N = 234
Sex (male)	176 (75%)
Age (years)	45 (35, 65)
Motor GCS	4 (1, 5)
Pupillary reactivity	
Both reactive	157 (67%)
One reactive	36 (15%)
None reactive	41 (18%)
DC	70 (30%)
EVD†	39 (17%)
Isolated TBI	54 (23%)
Intracranial Injury	
EDH	33 (14%)
ICH	48 (21%)
SDH	160 (68%)
contusion	140 (60%)
tSAH	154 (66%)
Extracranial Injury	
Abdomen	34 (15%)
Bone extremities	43 (18%)
Pelvis	26 (11%)
Skull	128 (55%)
Spine	49 (21%)
Thorax	100 (43%)
GOS	
Good Recovery	31 (13%)
Moderate Disability	67 (29%)
Severe Disability	65 (28%)
Dead/Vegetative	71 (30%)

*Data shown as median (interquartile range) or number (%); Abbreviations: GCS – Glasgow Coma Scale; GOS – Glasgow Outcome Scale; DC – Decompressive Craniectomy; EDH – Extradural Hematoma; EVD – External Ventricular Drain; ICH – Intracerebral Hematoma; SDH – Subdural Hematoma; tSAH – traumatic Subarachnoid Hemorrhage; TBI – Traumatic Brain Injury

†ICP was monitored in all patients using a pressure transducer. Patients with refractory ICP increases received an EVD to allow cerebrospinal fluid drainage and ICP reduction

**Fig. 2:**
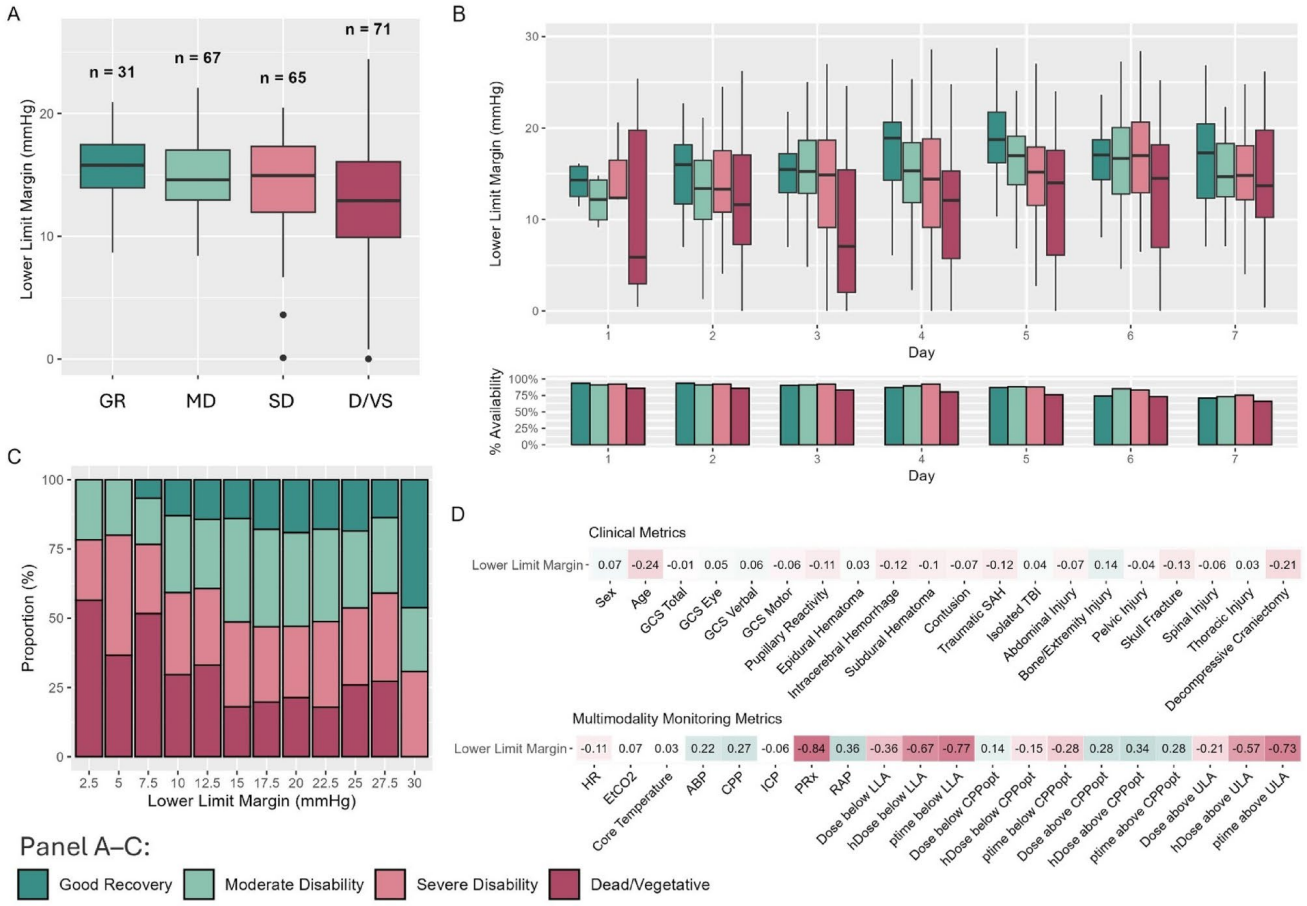
The association between the Lower Limit Margin, outcome, and clinical/multimodality monitoring metrics**.** Panel **A** displays overall averages of Lower Limit Margin depending on 6-month outcome, while Panel **B** displays the Lower Limit Margins depending on outcome throughout the first 7 days after injury. The bottom chart of Panel B displays the percentage data availability per outcome category per day. Overall, there were narrower margins in patients with worse outcomes, but these were highly dynamic throughout the stay. Panel **C** displays a histogram describing the different levels of day-to-day Lower Limit Margin values depending on outcome. In line with the boxplots, narrower Lower Limit Margins were distinctly more frequent in patients with worse outcomes. Panel **D** displays the correlations between Lower Limit Margin and clinical parameters (on top) or multimodality monitoring metrics (on the bottom). The resulting correlation coefficients are either colored red (for negative associations) or green (for positive associations), with the depth of color representing the strength of association. Only weak associations could be identified, with a weak negative association between broader Lower Limit Margin and older age. Conversely, when considering multimodality metrics, there were strong associations between the Lower Limit Margin and the extent of CPP below LLA.

Figure 2 describes the main results focusing on the first question, namely the assessment of the Lower Limit Margin as a prognostic marker. Overall (Fig. 2A; Table 2), there was a decrease in odds of unfavorable outcome with increasing Lower Limit Margin width (OR 0.59, CI 0.41–0.83, *p* = 0.003). These associations also remained relevant within some of the ordinal analyses (Table 2) but were overall weaker when adjusted for the relevant clinical parameters. Similarly, when assessed on a day-to-day basis (Fig. 2B, with the data-distribution described in Supplement A), a narrower Lower Limit Margin seemed to have a degree of linear association with worse outcome, across all studied days post-injury (with a linear decrease of − 3.53 mmHg (standard error 0.75) per outcome category). Of note, there was a larger interquartile range for the category dead/vegetative when compared to other categories, particularly for days 1 to 3. Figure 2C displays the hourly distributions of Lower Limit Margin depending on the outcome category, with very wide and very narrow margins being more represented in patients with better and worse outcomes, respectively. Overall, when considering overall averages, the Lower Limit Margin showed weak to no associations with clinical parameters but was more strongly associated with PRx (correlation of −0.84) as well as more frequent or severe deviations below the LLA (correlation coefficients of −0.77 and − 0.67 for ptime and hDose, respectively; Fig. 2D). To support the correlation analyses further, we performed a linear regression analysis between the regularly assessed clinical parameters and the Lower Limit Margin width (Table 3). Older age and DC were associated with narrower Lower Limit Margins, albeit with relatively small effect sizes corresponding to a decrease of 0.04 mmHg per year of age, and a decrease of 1.6 mmHg for patients who received a DC.

**Table 2:** Ordinal analyses

Metric	Model
	**Univariable logistic regression**
Lower Limit Margin	OR 0.59 (CI 0.41–0.83), *p* = 0.003
	**Sliding dichotomy**
Lower Limit Margin	OR 0.96(CI 0.90–1.02), *p* = 0.2
	**Proportional odds regression**
Lower Limit Margin	OR 0.89 (CI 0.84–0.94), *p* < 0.001

**Table 3:** Linear regression Analysis

Characteristic	Beta (95% CI)	*p*-value
Sex (male)	0.54 (−0.73, 1.8)	0.40
Age (years)	−0.04 (−0.07, −0.01)	0.006
Motor GCS	–0.16 (–0.44, 0.12)	0.30
Pupillary reactivity^†^	–0.58 (–1.6, 0.45)	0.30
DC	–1.6 (–2.8, −0.36)	0.011
EVD	0.29 (−1.2, 1.7)	0.70
Isolated TBI	0.42 (–0.89, 1.7)	0.50
Intracranial Injury		
EDH	0.42 (–0.89, 1.7)	0.50
ICH	–0.63 (–2.0, 0.70)	0.40
SDH	–1.3 (–2.5, −0.16)	0.027
contusion	–0.78 (–1.9, 0.33)	0.20
tSAH	–0.85 (–2.0, 0.30)	0.15
Extracranial Injury		
Abdomen	–0.98 (–2.5, 0.57)	0.20
Bone extremities	1.6 (0.20, 3.0)	0.025
Pelvis	–0.70 (–2.5, 1.1)	0.40
Skull	–0.88 (–2.0, 0.21)	0.11
Spine	–0.23 (–1.6, 1.1)	0.70
Thorax	0.10 (–1.0, 1.2)	0.90

The results of the regression analyses with lower limit margin as the dependent and the clinical parameters as the independent variables are shown describing the regression coefficients (Beta), the 95% confidence intervals, and the corresponding p-values.*

*Data shown as median (interquartile range) or number (%); Abbreviations: GCS – Glasgow Coma Scale; CI – confidence interval; DC – Decompressive Craniectomy; EDH – Extradural Hematoma; EVD - External Ventricular Drain; ICH – Intracerebral Hematoma; SDH – Subdural Hematoma; tSAH – traumatic Subarachnoid Hemorrhage; TBI – Traumatic Brain Injury

^†^both reactive vs. one reactive vs. none reactive

**Fig. 3:**
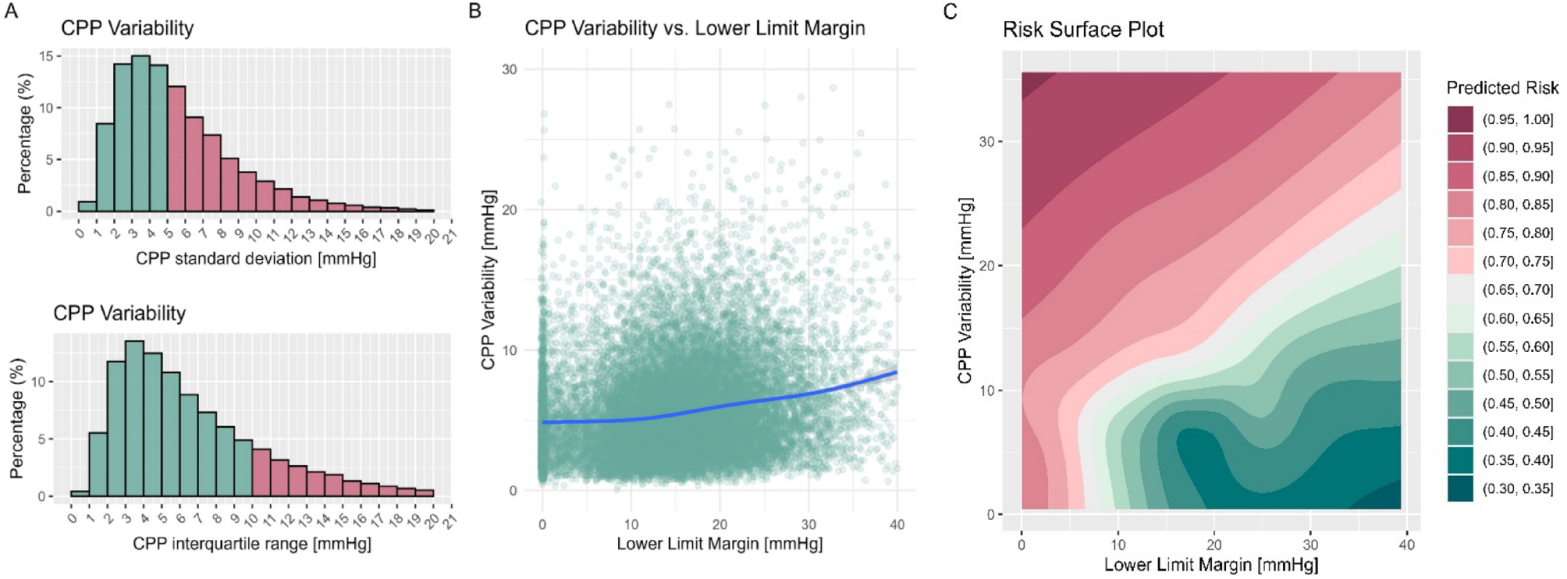
Association between the Lower Limit Margin, CPP variability, and outcome**.** Panel **A** displays the hourly distributions of CPP (quantified as standard deviation – i.e. short-term CPP variability, and interquartile range). The proportions with a variability above 5 mmHg standard deviation or above 10 mmHg interquartile range are colored red. Panel **B** displays the hourly distributions of Lower Limit Margin and short-term CPP variability. Of note, there are not distinct clusters of outliers or distinct patterns showing an association between Lower Limit Margin width and short-term CPP variability. The superimposed Locally Weighted Scatterplot Smoothing regression reveals only a minor increase in variability from 5 to 7 mmHg with increasing Lower Limit Margin Width. Lastly, panel **C** displays the risk surface plot – i.e., the predicted probability of unfavorable outcome as a function of different Lower Limit Margin and short-term CPP variability combinations. Overall, a distinctly asymmetric relationship can be identified with a more detrimental effect of narrow Lower Limit Margin and higher short-term CPP variability on outcome. The resulting effect is likely mediated by the increased time spent below the LLA in patients with narrow Lower Limit Margins and higher short-term CPP variability as described in the results section. Similarly, the association between very narrow Lower Limit Margins and worse outcome is likely mediated by the association to high PRx as described in Figure 2

Figure 3 describes the results assessing the relationship between the Lower Limit Margin and short-term CPP variability. Overall, patients spent 48% of their time with an hourly short-term CPP variability above 5 mmHg (Fig. 3A). When considering the variability in CPP and the Lower Limit Margin, no clear interaction could be identified visually (Fig. 3B). Lastly, Fig. 3C displays the predicted probability of unfavorable outcome as a function of both the Lower Limit Margin and short-term CPP variability, derived from a generalized additive model. The resulting risk surface demonstrates that the probability of poor outcome was highest when the Lower Limit Margin was narrow and short-term CPP variability was high. Conversely, increasing Lower Limit Margin width provides a “protective” effect against higher short-term CPP variability. When assessed using univariable mixed effects models, wider Lower Limit Margins were associated with less time spent below LLA (β −2.1, CI −2.2 to −1.9). Short-term CPP variability on its own, on the other hand, was not associated with changes in time spent below the LLA (β −0.04, CI −0.13 to −0.05). Conversely, when these metrics are added together to a mixed effects model, the effect of the Lower Limit Margin on the time spent below the LLA is amplified with higher short-term CPP variability (β = 0.15, *p* < 0.001, reflecting a significant interaction). This means to say that patients with narrow Lower Limit Margins spent significantly more time below the LLA if they also had a higher short-term CPP variability. Overall, the model explained 54% of the variance in the dependent variable (time below the LLA, *R²* = 0.536). In line with these results, the causal mediation analysis revealed a statistically significant average causal mediation effect (− 0.034, CI − 0.055 to − 0.010, *p* < 0.001), while there was no average direct effect (*p* = 0.08). There was a significant total effect of Lower Limit Margin width on functional outcome (*p* < 0.001), with 58.9% being mediated by the percentage time spent below the LLA.

## Discussion

In this exploration, we carried out an in-depth analysis of the recently introduced Lower Limit Margin, drawing attention to the importance of assessing the mechanism including different descriptors (i.e. the LLA and CPPopt). We demonstrated a consistent association between a narrow Lower Limit Margin and poor outcomes. Specifically, patients with narrower margins spent on average more time with CPP below the LLA, an effect which was, not surprisingly, potentiated by increased short-term CPP variability. These results indicate that the Lower Limit Margin should be considered as a dynamic marker of autoregulatory vulnerability to short-term CPP variability and may be critical in refining individualized cerebral perfusion targets strategies in patients with severe TBI.

To date, the management of CPP after TBI is largely based on fixed CPP targets [24]. While thresholds of 60–70 mmHg remain guideline-recommended, there is increasing consensus that individualized CPP targets, accounting for real-time cerebrovascular reactivity, may be superior for improving cerebral physiology [25] and outcome prediction [13]. CPPopt and LLA represent two key autoregulation-based metrics, but optimal strategies for targeting these parameters remain debated, with no study establishing the superiority of either target. The present study provides evidence that the absolute distance between CPPopt and LLA is a critical marker of ‘autoregulatory resilience’ to short term variations in CPP. Our findings also emphasize the dynamic, patient-specific nature of the Lower Limit Margin throughout the ICU course. The width of the Lower Limit Margin differed vastly between patients and over the ICU course, with no clear association to clinical markers of disease severity. This aligns with prior physiological studies demonstrating that the autoregulatory range may be substantially compressed in acute brain injury and may vary depending on the type of injury [26].

The mechanistic plausibility of the Lower Limit Margin as a marker of vulnerability was bolstered by several lines of analysis. First, the Lower Limit Margin was strongly correlated with PRx and time spent below the LLA – two well-established proxies of impaired autoregulation and perfusion inadequacy – when considering overall averages. Second, causal mediation analysis suggested that more than half of the effect of Lower Limit Margin width on outcome is mediated through time spent below the LLA, further supporting its role as a mediator of impaired autoregulatory burden. Lastly, generalized additive models revealed that the combination of a narrow Lower Limit Margin and high short-term CPP variability was associated with the worst outcome, highlighting the importance of a nuanced evaluation of both autoregulatory reserve and hemodynamic instability.

From a clinical standpoint, several key insights emerge. First, we observed marked hour-to-hour variability in CPP, with nearly 50% of the monitored time exceeding a standard deviation of ± 5 mmHg. Consequently, it is questionable whether such narrow targets are even achievable from a purely practical standpoint. While CPPopt has become central to autoregulation-guided CPP management, our findings offer further support [4, 5] that applying a fixed buffer around this value, without accounting for the patient’s individual Lower Limit Margin, is an oversimplification that may carry unintended risks. Importantly, our data demonstrates that the width of the Lower Limit Margin fundamentally alters the physiological significance of short-term CPP variability. Patients with a wide Lower Limit Margin exhibited greater tolerance to CPP excursions, indicating that rigid adherence to a narrow CPPopt window may not be necessary in this group. In contrast, patients with a narrow Lower Limit Margin are disproportionately vulnerable to brief deviations in CPP, underscoring the need for tighter hemodynamic control in this population. In such cases, even small reductions in CPP are more likely to cross below the LLA, associated with impaired autoregulation and potential hypoperfusion. Crucially, the expected time spent below the LLA is governed not only by the interaction between absolute Lower Limit Margin width and short-term CPP variability, but certainly also by the proximity of the occurring variability to the different autoregulation limits. This means to say that even if short-term CPP variability occurs, when such variability occurs beyond CPPopt away from the LLA, this may not be detrimental as shown in previous research [13].

These observations should shift the paradigm: the traditionally accepted ± 5 mmHg range around CPPopt may not be universally protective and may be inadequate, or even harmful, for patients with limited autoregulatory reserve (reflected by a narrow Lower Limit Margin). Similarly, assessing only the LLA may be inadequate, since it does not provide the clinician with sufficient information regarding the autoregulatory range and to what degree changes in CPP are acceptable from a physiological standpoint. Therefore, individualized assessment of the Lower Limit Margin should inform not only the target CPP but also the permissible degree of variability around it. This view reinforces the need for a dynamic, resilience-informed approach to CPP management considering CPPopt, LLA, and the Lower Limit Margin, rather than static thresholds.

### Limitations

A key limitation of this study is the single-center design, potentially limiting wider generalizability despite the guideline-based management. Importantly, whether CPPopt-based management is carried out remains up to the treating physician, even though it is suggested as part of the routine treatment regimen [27]. In addition, even if PRx may be, under certain circumstances, improved by adjusting CPP based on the autoregulation-derived targets, no currently available intervention adjusts the underlying relationship between PRx and CPP (and consequently the Lower Limit Margin) [28]. Different interventions (including CO2 management, cerebral temperature), however, may inadvertently affect the relationship between CPP and cerebral blood flow [6, 29]. Additionally, the measured short-term CPP variability may reflect both physiological instability and treatment interventions (e.g., osmotherapy, vasopressors). Disentangling endogenous vs. iatrogenic variability could not be addressed in this analysis with the available data. We chose to keep patients who received DC or had an inserted external ventricular drain to allow for a better representation of ICU admitted patients within a trauma-center. In certain cases these may inadvertently violate the assumptions set for the calculation of PRx (i.e. changes in ICP introduced by mechanisms other than changes in blood pressure slow-waves such as due to using the external ventricular drain for cerebrospinal fluid drainage; as well as the necessity for these slow waves to be translated into changes in ICP, an assumption which may be violated in patients after DC with very high compliance). However, we believe that in the context of the presented statistical analyses these effects would only dilute the, expected, associations found in the data, through introduction of artefactual values of PRx, and thus do not detract from the main messages of this paper.

Moreover, CPPopt, LLA and their derived metrics are derived from a large period of up to 8 h of continuous monitoring data. Consequently, time-varying (physiological or iatrogenic) shifts in the CPP–PRx relationship within a window are averaged rather than explicitly captured. This temporal smoothing can influence both the estimates and their interpretation, a limitation inherent to studies using continuous CPPopt/LLA-derived metrics that should be borne in mind when drawing conclusions. Lastly, it is important to note that PRx, as well as the many other cerebrovascular autoregulation metrics, only permit approximations of the true state of cerebrovascular autoregulation [30]. Lastly, there are various other parameters and secondary consequences of brain injury that were not explored in this analysis, but undoubtedly also affect outcome.

## Conclusion

This study identifies the Lower Limit Margin as a robust, dynamic marker of autoregulatory reserve in patients with TBI. A narrower Lower Limit Margin is associated with greater exposure to CPP levels below the LLA, particularly in the presence of high short-term CPP variability. Future work should explore whether real-time tracking of the Lower Limit Margin and adaptive modification of CPP targets may mitigate autoregulatory insults and ultimately improve patient outcomes. By shifting from a fixed-threshold or single-target (i.e., CPPopt or LLA) to a resilience-informed approach to CPP management, we may ultimately move closer to a better individualized strategy for CPP management in TBI.

## Supplementary Information

Supplementary Material 1

## Data Availability

Postprocessed data is available upon reasonable request to the corresponding author.
