# Supplementary material for: The lower limit margin: a determinant of autoregulatory sensitivity to changes in cerebral perfusion pressure
Source: Crit Care. 2025 Oct 27;29:455. doi: 10.1186/s13054-025-05686-z (PMC12560507; doi:10.1186/s13054-025-05686-z)

**Supplement A: CPP Data Coverage.** The CPP data coverage is shown in form of boxplots (length – A; stratified by outcome GOS 1-3 vs. 4-5) and density plots (frequency of data relative to day post injury or onset of monitoring (with day 0 being the day of the initial injury) - B; stratified by GOS category). The median number of hours available was 157 (IQR 88-279). No difference in length of monitoring could be identified when considering patients with unfavorable vs. favorable outcomes (i.e. GOS 1-3 vs. GOS 4/5) with median 167 (IQR 94-297) and 150 (IQR 83-275) hours available respectively (p=0.3). The vast majority was acquired within the first week of injury with similar distributions throughout the hospital stay irrespective of GOS category.


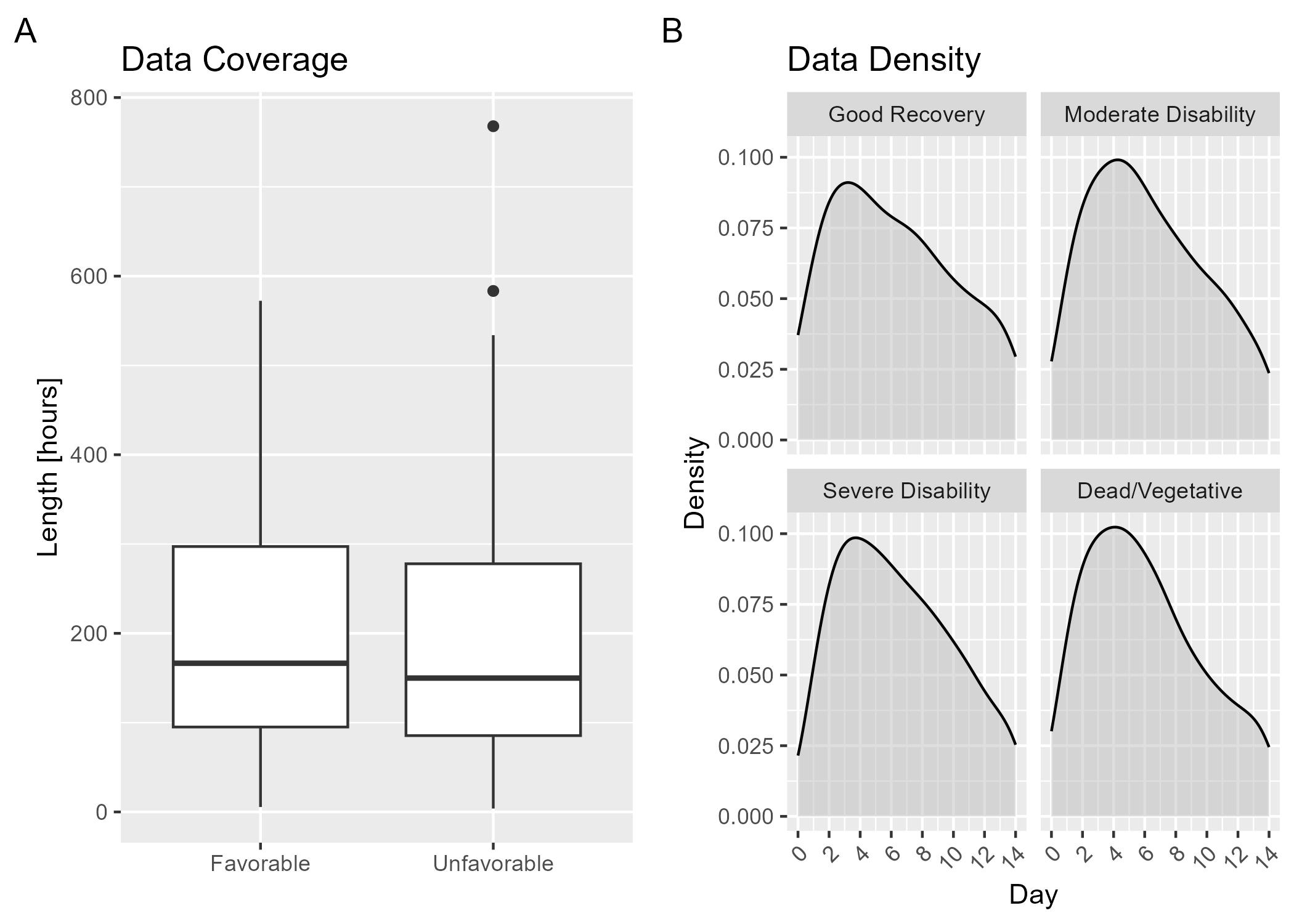

Supplement: Supplementary file 1 — Supplementary Material 1 [file 13054_2025_5686_MOESM1_ESM.docx]
